# Sexual Inequality in Tuberculosis

**DOI:** 10.1371/journal.pmed.1000199

**Published:** 2009-12-22

**Authors:** Olivier Neyrolles, Lluis Quintana-Murci

**Affiliations:** 1 Centre National de la Recherche Scientifique, Institut de Pharmacologie et de Biologie Structurale, Toulouse, France; 2 Université de Toulouse, Université Paul Sabatier, Institut de Pharmacologie et de Biologie Structurale, Toulouse, France; 3 Institut Pasteur, Human Evolutionary Genetics, CNRS, URA3012, Paris, France

## Abstract

Olivier Neyrolles and Lluis Quintana-Murci review the evidence on why tuberulosis notification is twice as high in men as in women in most countries.

**Summary Points**

- In most countries, tuberculosis (TB) notification is twice as high in men as in women.
- Although there is clear evidence that socioeconomic and cultural factors leading to barriers in accessing health care may cause undernotification in women, particularly in developing countries, biological mechanisms may actually account for a significant part of this difference between male and female susceptibility to TB.
- The role of biological gender has been determined in a number of infectious and noninfectious diseases. However, there is an absence of information on the role of biological gender in TB.
- Thus, investigations should be conducted to clearly understand the role of sexual hormones, sex-related genetic background and genetic regulations, and metabolism, among other factors, in susceptibility differences between men and women.
- This research may help not only to fully understand the obviously biased gender distribution among TB cases, but also to better adapt future intervention strategies at the community level. In this review, we expand on the various issues relating to TB notification and gender bias.

## The Sex Bias in TB Cases May Be of Biological Significance

**Figure 1:**
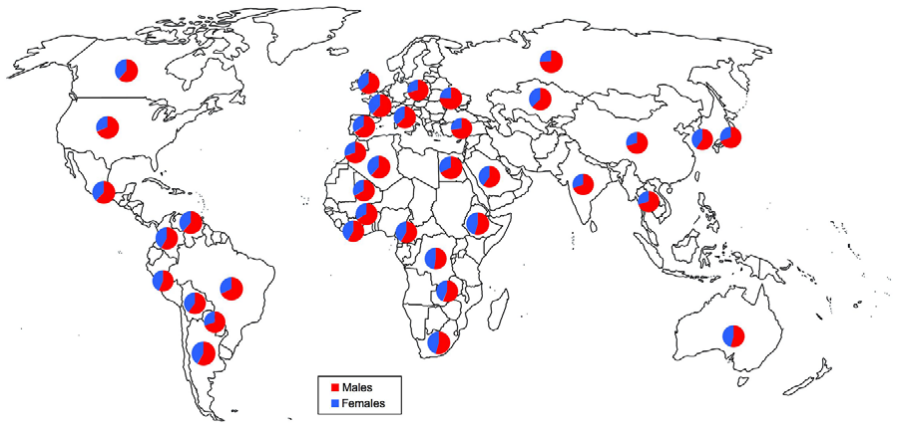
Sex distribution for new smear-positive TB case notification in 2007 in various countries [1].

**Figure 2:**
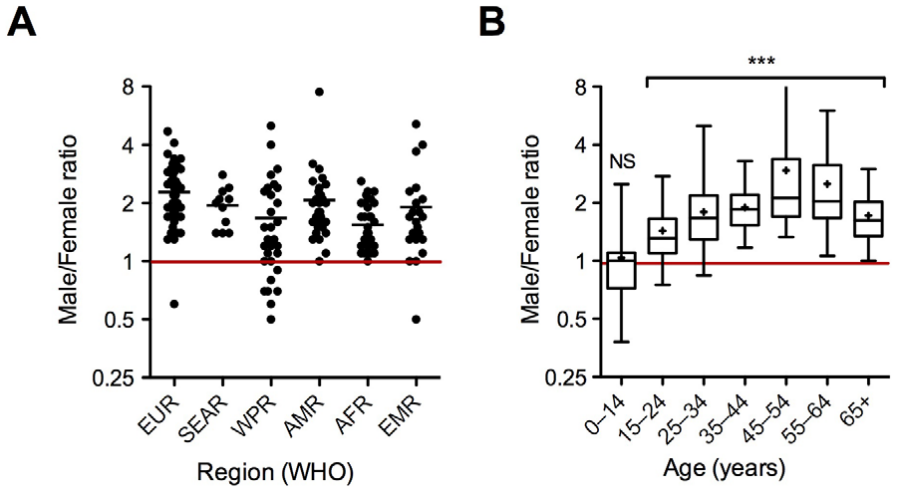
Regional (A) and age (B) distribution of the male/female ratio for new smear-positive TB cases in 2007. (A) Dot-plot in which each dot corresponds to a country. EUR, Europe; SEAR, Southeast Asia; WPR, Western Pacific; AMR, the Americas; AFR, Africa. The bar indicates the mean. (B) Box plot showing the 25th and 75th percentiles, together with the median, with whiskers showing the minimum and maximum male/female ratio for new smear-positive TB cases in 2007 in the Americas. Crosses indicate means. Data were compared with the male/female ratio expected under the hypothesis of neutrality (1, red line) and were analyzed with Mann-Whitney tests (***, *p*<0.0001) [1]. NS, not significant.

Tuberculosis (TB) claims over 1.7 million lives throughout the world each year according to the most recent World Health Organization (WHO) report [1]. Men seem to be more affected than women, with a male/female ratio of 1.9±0.6 for the worldwide case notification rate (Box 1; Figure 1) [1]. In some countries this ratio may reach values as high as 3 (4.7 in Armenia for instance), but ratios below 1 are extremely rare and mostly correspond to very small populations of patients [1]. This excess of male pulmonary TB cases is seen in all regions of the world, and in almost all countries (Figure 2A), at least in non–HIV-infected patients. It is also seen in adults of all ages, but does not seem to apply to children and young adolescents (Figure 2B).

**Box 1. Six Key Papers on Sex Bias in TB**

- Grossman CJ [13]. A thorough and early review on the links between the endocrine and the immune systems
- Naugler WE, Sakurai T, Kim S, Maeda S, Kim K, et al. [49]. An in-depth investigation of the molecular mechanisms through which estrogen inhibits IL-6 production and protects females from hepatocarcinoma. May inspire similar research in the TB field
- Yamamoto Y, Tomioka H, Sato K, Saito H, Yamada Y, et al. [23]. The first animal model-based report on differential susceptibility of males and females to a mycobacterial infection
- Hamid Salim MA, Declercq E, Van Deun A, Saki KAR [11]. One of the best examples of a population-wide prevalence survey concluding that there is a male/female bias in susceptibility to TB
- Borgdorff MW, Nagelkerke NJD, Dye C, Nunn P [10]. A meta-analysis of nearly 30 reports on notifications and prevalence data concluding that male/female ratio in TB is not due to differential access to health care in most cases
- Fortin A, Abel L, Casanova JL, Gros P [30]. A thorough review on current knowledge and future perspectives in genetics of mycobacterial infections

As case notification is a complicated process, beginning with the recognition of initial symptoms and followed by clinical diagnosis and reporting, this indicator necessarily combines various factors, including, for example, differences in both susceptibility and exposure, help-seeking behavior, and access to health care services. Several reviews have discussed the possibility of undernotification of women due to greater difficulties in gaining access to clinics and in obtaining a timely diagnosis and treatment, particularly in developing countries [2]. Other confounding factors, such as smoking, alcohol and drug use, exposure to indoor dusts and air pollution, as well as the poor quality of sputum samples collected from women in some regions, may influence the sex bias observed in patients with TB [3]–[8].

The case notification rate therefore may not reveal the many facets of inequality between male and female patients with TB. However, the poorer access of female patients to health care does not account for the higher incidence of detection on the basis of positive smears in a number of situations, including low-income countries [9]. A recent multicentre case-control study conducted in three West African countries concluded that male sex is indeed a risk factor for TB, independent of other factors examined; in this case multivariate analysis of environmental and host-related factors found a male/female ratio of 2.03 among patients with TB (versus 1.12 in the contact population, *p* = 0.02) [5].

Unlike studies based on case notification, large, systematic prevalence surveys can provide us with information about genuine biological differences in susceptibility to TB between men and women, if indeed there are any. A prime example is provided by a large prevalence survey conducted in Bangladesh, in which more than 260,000 individuals (51% males) were visited in a house-to-house survey designed to detect cases of suspected TB, for possible subsequent confirmation by smear observation [10]. An excess of cases in males, with a sex ratio of 3∶1 (48 males and 16 females with confirmed TB), was observed, even if confounding factors, such as income, awareness, and stigma were taken into account [11].

Leaving aside sociocultural biases, biological factors leading to differences in resistance to infection/disease between men and women may account, at least in part, for the worldwide excess of male pulmonary TB cases detected by case notification and epidemiological surveys. Surprisingly, this topic has been largely ignored in scientific and medical investigations, despite the importance of dissecting the biological processes underlying the observed differences between the sexes to our understanding of the mechanisms involved in host susceptibility to TB in the general population.

The human population as a whole is actually highly resistant to TB, with “only” 5% to 10% of exposed individuals going on to develop the disease during their lifetime [12]. Many factors, including the virulence of the infecting strain, and the nutritional status, hygiene, age, ethnic and genetic background, immunosuppression status, and, possibly, sex of the infected host, may account for the greater susceptibility of individuals developing the disease than of the remaining healthy population. However, several other, more specific biological sex-related factors may render men even more susceptible to pulmonary TB than women: sex steroid hormones, the genetic makeup of the sex chromosomes, and sex-specific metabolic features. These factors are, of course, interconnected in real life, but for practical reasons we will review them independently.

## Sex Steroids and the Antimycobacterial Immune Response

The effects of sex steroid hormones on the immune response to infection and other noninfectious immune disorders, and, more generally, the interplay between the endocrine and the immune systems, are widely documented in humans and animal models [13]–[16]. Simple experiments in castrated and hormone-reconstituted animals can reveal the influence of sex hormones on immune functions. For example, it has been reported that androgen deprivation due to the castration of male mice leads to an increase in the absolute number of T lymphocytes in the peripheral lymph nodes and an increase in the proliferation of these cells following antigen recognition [17].

Other examples are provided by reports showing that estradiol enhances macrophage activation [18], and that invariant natural killer T (iNKT) cells from female mice produce more interferon-gamma (IFNγ) than do the cells from male mice in response to in vivo challenge with the iNKT cell ligand α-GalCer [19]. Female castration or genetic ablation of the estradiol receptor impairs IFNγ production by these cells, the normal phenotype being restored by estradiol injection. These results illustrate the general, but probably simplistic perception of estradiol as an immunity-sustaining or immunity-enhancing mediator, and of testosterone as a mediator inhibiting the immune response [20].

Surprisingly, very few studies have considered the role of sex steroids in host protection and susceptibility to TB in humans or animals. The male gender bias in TB detection rate may involve sex hormones as it becomes apparent after sexual maturity (Figure 2B). This suggests that sexual hormones may play a part in protection/susceptibility to TB. The possible role of sex steroids in TB is also strengthened by the fact that the progression-to-disease and mortality rates are higher in females during their reproductive years, after which such rates turn again to be higher in men (reviewed in [2],[21]).

Early reports for other mycobacterial infections shed some light on this issue. Female mice are more resistant to infection with bacteria of two species related to *Mycobacterium tuberculosis*: *M. intracellulare* and *M. marinum* [22],[23]. The treatment of females or castrated males with testosterone increases their susceptibility to *M. marinum*, and it has been suggested that the higher susceptibility of males to both these infections may be due to differences between the sexes in terms of innate resistance mechanisms mediated by host phagocytes. The role of estradiol in mycobacterial infections has also been investigated in other experimental settings. Estradiol treatment abolishes the greater susceptibility of ovariectomized mice to *M. avium*, with estradiol again appearing to act in synergy with IFNγ to impair mycobacterial growth [24].

Similar results have been obtained for other intracellular pathogens, such as *Coxiella* [25], and the parasite *Leishmania*. As for TB, the incidence of visceral leishmaniasis seems to be higher in males, and there is experimental evidence to suggest that the protective Th1 response associated with IFNγ production is stronger in females, at least partly because of the action of estrogens [26]. This potential role of estrogens is not surprising, as estrogen has been shown to increase the activity of the IFNγ gene promoter [27]. Levels of steroid hormones vary not only between the sexes, but also with age and physiological state (e.g., menstrual cycles and gestation). The influence of these variations of sex hormone levels on resistance or susceptibility to TB remains to be investigated.

## Is TB Associated with a Sex-Specific Genetic Architecture?

It is now widely accepted that host genetic factors play a major role in determining differential susceptibility to infection and disease outcome in humans [28],[29]. Most studies in the context of TB have investigated the role of specific candidate genes, chosen on the basis of the effects of their murine orthologs on the response to experimental mycobacterial infections or the known biology of the disease. Despite the fact that the quality of these studies varies greatly, genetic variation in an increasing number of genes (e.g., *NRAMP1*, *HLA* class II, *VDR*, *MAL/TIRAP*, *DC-SIGN*, *MCP-1*, *TLR8*) has been found to be associated with complex susceptibility to pulmonary TB (reviewed in [29],[30]).

Unlike candidate-gene studies, in which the choice of the genes to be tested may be arbitrary, genome-wide linkage analyses are more systematic and suitable for the identification of loci with a substantial effect on disease phenotype (i.e., major susceptibility loci). A few genome-wide linkage analyses have been performed to date, and the results are not always consistent among studies. Early studies provided evidence for linkage on Chromosomes 15q and Xq in African families [31], and 11q and 20p in Brazilian families [32]. However, these studies did not provide evidence for the existence of a major susceptibility locus. The first major locus identified by genome-wide linkage analyses was recently mapped to 8q12-q13, which houses at least one major gene that confers predisposition to pulmonary TB in adults with a dominant mode of inheritance [33]. The precise genes and variants within this region actually involved in susceptibility to TB remain to be identified.

Some of the reported associations remain to be confirmed, but we now need to consider how host genetic variation at these loci (or other loci yet to be identified) results in sex-specific differences in TB incidence. Intuitively, it would seem reasonable to assume that the sex chromosomes make some contribution. The first genome-wide linkage analysis of TB identified a region on Xq for which there was suggestive, but not significant, evidence for linkage to TB [31]. However, this finding has not been replicated by any other genome-wide linkage study, and no association between any gene located on Xq and complex susceptibility to TB has yet been reported.

A recent association study screening variation at 18 genes involved in the Toll-like receptor (TLR) pathway identified four polymorphisms in the *TLR8* gene, which is located on chromosome X (Xp22), which seemed to be associated with complex susceptibility to pulmonary TB in an Indonesian cohort [34]. All four of these polymorphisms are in perfect linkage disequilibrium, and one (rs3764880) is a missense variant (Val1Met) that could indeed be the functional polymorphism associated with the disease. When performing the tests separately for men and women, the authors observed a strong association between the rs3764880 allele A (Met) and susceptibility to TB that was restricted to men.

This association was subsequently replicated in a large, independent cohort from Russia, suggesting that there may be a genuine effect. The frequency of the “susceptibility” allele (rs3764880 allele A) is very different in the Indonesian (∼30%) and Russian (∼80%) populations. This observation suggests that the relative effect of this allele on susceptibility to TB may differ in the two populations, owing to differences in TB exposure, the virulence of the bacteria, and differences in the genetic make-up of the two human populations, for example. The association observed between X-linked variation at the TLR8 gene locus and complex susceptibility to TB is consistent with sex-specific effects on the genetic architecture of TB [34].

Indirect evidence for a possible role of the X chromosome in susceptibility to TB has also been provided by studies of Mendelian susceptibility to mycobacterial diseases (MSMD). Causal mutations in an X-linked gene (*NEMO*) and in two candidate regions on Xp11.4–Xp21.2 and Xq25–Xq26.3 have been identified in patients suffering from MSMD, some of whom also have presented TB [35],[36]. These studies suggest that variation in the X chromosome may be involved in the genetic predisposition to TB, from both Mendelian and complex genetic perspectives [30].

However, much remains to be done. A better appreciation of the extent to which sex-specific genetic effects lead to differences in the prevalence of TB between men and women requires further large-scale genetic studies, involving large cohorts of clinically well-defined TB cases and appropriate controls, stratified by sex. In particular, sex-specific genetic effects may not be restricted to the obvious case of sex chromosomes. Autosomal DNA sequences do not differ between men and women, but differences in gene regulation between the sexes have been documented (reviewed in [37]). Thus, studies on sex-specific differences in gene regulation in the context of TB should help to delineate the basis of the phenotypic sexual dimorphism observed for susceptibility to this major disease.

## Sex, Nutrition, and TB

Sex-specific features of nutrition and metabolism may also be associated with susceptibility or resistance to *M. tuberculosis*. Iron, for instance, is a crucial component of several enzymes and redox systems in mycobacteria, as in all living organisms. The extrusion of iron from the microbial vacuole has long been recognized as an innate immune system mechanism, conserved throughout evolution, for host phagocyte control of various intracellular pathogens, including mycobacteria [38]. Iron deficiency is common in women from developing and industrialized countries [39]. It remains unclear whether anemia is correlated with greater resistance to TB in humans. However, experimental evidence from animals suggests that iron overload increases permissiveness to *M. tuberculosis* considerably, both in vivo [40] and in vitro [41].

Major differences have also been found between the sexes in terms of fat metabolism. These differences may influence susceptibility to infectious disease, and to TB in particular [42]. Other nutrients and cofactors, such as vitamin D [43],[44], play an important role in antimycobacterial immunity, and their possible role in sex-related aspects of the immune response to *M. tuberculosis* remains to be established. Again, the effect of nutrients and nutritional status on differential susceptibility to TB may rely on sex hormones, as recently exemplified by the estrogen/vitamin D synergy in resistance to experimental autoimmune encephalomyelitis [45].

Finally, other yet unsuspected gender-related features may render men more susceptible to *M. tuberculosis* than women. For instance, it is well known that men and women present important differences in the anatomy and physiology of the upper airway and respiratory tract [46]. These differences include structural and functional differences (e.g. oropharyngeal length and ventilatory functions) as well as histological differences (e.g., the amount and distribution of the fat around the pharynx and along the upper airway). Some of these features are indeed influenced by sex steroids and aging, and whether they play a part in differential susceptibility to airborne infections, including TB, has yet to be evaluated.

## Conclusion

Large prevalence surveys have suggested that the sex bias observed in pulmonary TB cases may result partly from genuine biological differences in male and female susceptibility to *M. tuberculosis* infection or the development of TB disease. This finding would not be particularly surprising, as many studies in humans and experimentally infected animals have established clear links between sex-specific factors, including steroid hormones and genetic variants, and the differential susceptibility of males and females to a number of other infectious and noninfectious diseases. In particular, gender bias among pulmonary microbial diseases is not restricted to TB, and important sex differences in the incidence and severity of a number of respiratory tract bacterial infections have been reported in the literature [47]. As a selected example, it has been shown that men have a 4-times higher risk of developing nosocomial *Legionella pneumophila* infection than women [48].

**Figure 3:**
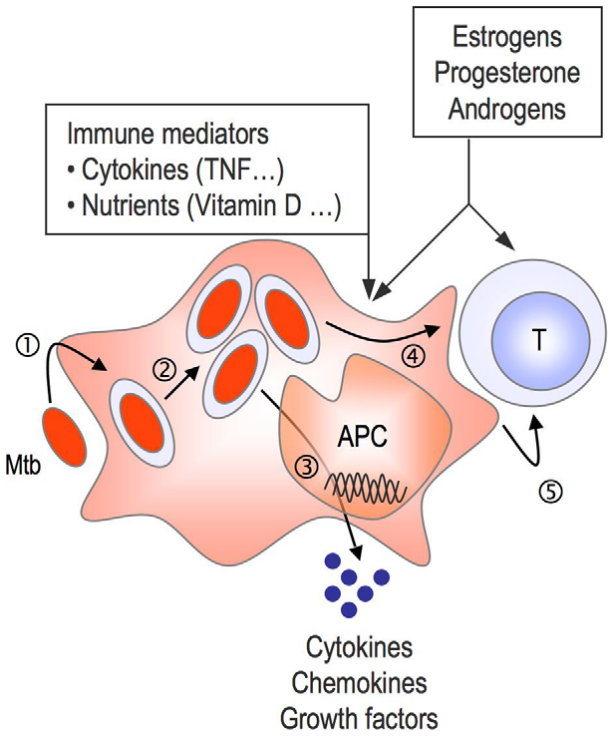
Do sex steroids influence antimycobacterial immunity? Both innate immune cells (monocytes, macrophages, and dendritic cells) and T cells express specific receptors for steroid hormones, at least a fraction of them [15]. Future experimental work may assess whether sexual hormones, alone or in combination with other immune mediators, influence *M. tuberculosis* (Mtb) entry (1) and intracellular trafficking and survival (2) in host phagocytes and antigen-presenting cells (APC), the secretion of cytokine and other factors by infected cells (3), antigen presentation (4), and T cell development (5).

Only 5% to 10% of individuals exposed to *M. tuberculosis* develop TB, and up to 70% of those who do develop the disease are male. In other words, the human population as a whole is remarkably resistant to *M. tuberculosis*, but women seem to be even more resistant to the bacillus than men. So, why do only a minority of individuals, other than patients with HIV/AIDS, fail to control infection? Why are women less likely to develop TB than men? Why are some women more resistant to TB than other women exposed to a similar extent? Field research consortia including not only microbiologists, immunologists, and human geneticists, but also epidemiologists and sociologists, should be established to unravel the many faces of sexual inequality in TB, and to decipher the delicate mechanisms involved in natural and sex-associated resistance to TB (Box 2; Figure 3). Such work would facilitate the design of future intervention strategies for combating the disease and the development of useful tools for evaluating prognosis and protection in future clinical trials.

**Box 2. Key Research Actions on Sex Bias in TB**

- Parallel and homogeneous epidemiological surveys in human populations from different geographic and ethnic backgrounds to dissect simultaneously the various factors possibly contributing to the sex bias in TB in the most exhaustive manner, including:
  - Sociocultural components: income, stigmatization, awareness, etc.
  - Behavioural components: smoking, alcohol and drug abuse, exposure to toxic dusts at the work place, dietary differences, etc.
  - Biological components: sex hormones, genetic background
- Detailed follow-ups of sex hormone profiles in men and women presenting TB, as well as in the corresponding healthy contacts exposed to the same environmental pressures
- Development of an appropriate animal model that mimics the sex bias observed in TB in humans for subsequent in vivo dissection of the influence of sex hormones in castrated and hormone-reconstituted animals on immune response to *M. tuberculosis* and disease outcome
- Development of suitable in vitro cell models to investigate the influence of sex hormones and immune modulators (cytokines and nutrients such as iron, vitamin D, etc.) on the immune response to *M. tuberculosis* (see Figure 3)
- Genome-wide association studies in populations from diverse geographic areas, involving large cohorts of clinically well-defined TB cases and appropriate controls, stratified by sex
- Genome-wide gene expression profiling in different in vitro and ex vivo biological settings (e.g., monocyte-derived phagocytes, blood samples, lung biopsies, broncho-alveolar lavages) from male and female TB patients and relevant controls

## Supporting Information

Alternative Language Abstract S1 French translation of the abstract by ON.
(0.04 MB DOC)

Alternative Language Abstract S2 Spanish translation of the abstract by LQM.
(0.04 MB DOC)
