# Supplementary material for: Sexual Inequality in Tuberculosis
Source: PLoS Med. 2009 Dec 22;6(12):e1000199. doi: 10.1371/journal.pmed.1000199 (PMC2788129; doi:10.1371/journal.pmed.1000199)
Supplement: Alternative Language Abstract S1 — French translation of the abstract by ON. (0.04 MB DOC) [file pmed.1000199.s001.doc]

**Dans la plupart des pays, la tuberculose touche deux fois plus d’hommes que de femmes. Bien que la preuve ait été faite que des facteurs socio-économiques et culturels créant des barrières dans l’accès aux soins peuvent conduire à une sous-notification des femmes, en particulier dans les pays en développement, des mécanismes biologiques pourraient bien aussi contribuer pour une part non négligeable à cette différence de susceptibilité à la tuberculose entre les hommes et les femmes. Le rôle du genre “biologique” et de toutes ses composantes (génétiques, hormonales, métaboliques *etc*) a été bien déterminé dans un grand nombre de maladies infectieuses et non-infectieuses. Pourtant le rôle du genre biologique dans la tuberculose a été peu ou pas étudié. Ainsi il faudrait conduire des recherches pour mieux comprendre le rôle des hormones sexuelles, et des différences génétiques et métaboliques, entre autres, dans la susceptibilité différentielle des hommes et des femmes à la tuberculose. Ceci permettrait non seulement de comprendre la distribution biaisée du genre parmi les cas de tuberculose, mais aussi d’aider à adapter les futures stratégies d’intervention au niveau de la communauté. C’est ce que nous souhaitons discuter ici.**
