# Supplementary material for: Sexual Inequality in Tuberculosis
Source: PLoS Med. 2009 Dec 22;6(12):e1000199. doi: 10.1371/journal.pmed.1000199 (PMC2788129; doi:10.1371/journal.pmed.1000199)
Supplement: Alternative Language Abstract S2 — Spanish translation of the abstract by LQM. (0.04 MB DOC) [file pmed.1000199.s002.doc]

En la mayoría de países, la incidencia de la tuberculosis es dos veces más importante en hombres que en mujeres. Aunque factores socioeconómicos y culturales como el acceso a servicios médicos explica en parte que un menor número de mujeres sean diagnosticadas, en especial en países en vías de desarrollo, causas biológicas pueden explicar la diferencia en susceptibilidad a la tuberculosis entre hombres y mujeres. La influencia del sexo biológico y todo lo que conlleva (genética, hormonas, metabolismo, *etc*) ha sido ya demostrada en diferentes enfermedades infecciosas y no-infecciosas. Sin embargo, la literatura científica y médica con respecto a la influencia del sexo biológico en la susceptibilidad a la tuberculosis es prácticamente inexistente. En el mundo científico, y mas concretamente en el estudio de las enfermedades infecciosas, hay una necesidad evidente en estudiar como los cambios hormonales entre los dos sexos, el /background/ genético y la regulación de la expresión génica asociados al sexo, y el metabolismo, entre otros factores, pueden jugar un rol importante en la susceptibilidad a la tuberculosis. Estudios de este tipo no solo serian importantes para entender las razones de una distribución diferente de la tuberculosis entre hombres y mujeres, pero también serian de una importancia mayor para adaptar en un futuro próximo estrategias de intervención médica a nivel de la comunidad.
